# A sustainable natural clam shell derived photocatalyst for the effective adsorption and photodegradation of organic dyes

**DOI:** 10.1038/s41598-022-06981-3

**Published:** 2022-02-22

**Authors:** Ting Qu, Xinxin Yao, Gary Owens, Liangjun Gao, Hailong Zhang

**Affiliations:** 1 National Engineering Research Center for Marine Aquaculture, Institute of Innovation & Application, Zhejiang Ocean University, Zhoushan, 316022 Zhejiang China; 2 Zhejiang Key Laboratory of Petrochemical Environmental Pollution Control, National-Local Joint Engineering Laboratory of Harbor Oil and Gas Storage and Transportation Technology, School of Petrochemical Engineering and Environment, Zhejiang Ocean University, Zhoushan, 316022 Zhejiang China; 3 Environmental Contaminants Group, Future Industries Institute, University of South Australia, Mawson Lakes Campus, Mawson Lakes, South Australia 5095 Australia; 4 College of Naval Architecture and Mechanical-Electrical Engineering, Zhejiang Ocean University, Zhoushan, 316022 Zhejiang China

**Keywords:** Environmental sciences, Environmental chemistry

## Abstract

In response to an increasing desire for modern industries to be both green and sustainable, there has been increasing research focus on the reutilization of natural waste materials to effectively remove and degrade toxic wastewater effluents. One interesting food industry waste product is clam shell. Here a new photocatalytic nanomaterial derived from marine clam shells was successfully prepared and characterized. Thereafter the material was applied for the removal of two target dyes from aqueous solution, where the effect of both catalyst dose and initial dye concentration on adsorption and photocatalysis was investigated. The maximum adsorption capacities of methylene blue (100 mg/L) and Congo red (500 mg/L) were 123.45 mg/g and 679.91 mg/g, respectively, where adsorption followed pseudo second order kinetics predominantly via a chemical adsorption process. The photodegradation removal efficiencies of the two dye solutions under visible light irradiation were 99.6% and 83.3% for MB and CR, respectively. The excellent degradation performance in a mixed dye solution, with strong degradation capability and low cost, demonstrated that the clam shell catalyst material was a good candidate for practical field remediation of dye contaminated wastewater.

## Introduction

While water is essential for all life, the rapid growth in modern human civilizations globally, concurrent with vigorous and rapid industrialization has resulted in significant pollution of water resources through the discharge of waste materials, especially by the dye industry. Water resource contamination by dye wastewaters is becoming increasingly serious^1^. Methylene blue (MB), Congo red (CR) and many other organic dyes are widely and routinely used in large quantities by various dye industries. These dye molecules have a complex molecular structure containing many diverse functional groups such as amino, hydroxyl and the benzene ring^2^, which while giving the dye strong coloring ability also results in strong toxicity and poor biodegradability^3^. These latter features are of concern because the efflux of large concentrations of dyes into the natural environment may thus lead to environmental harm. Hence, development of new materials to treat dye wastewater is urgently required to mitigate environmental pollution. While many non-destructive methods, such as adsorption, membrane filtration, and precipitation, have previously been successfully employed to remove dye from wastewater, the disadvantage of non-destructive methods are that do they nor destroy the dye but merely concentrate the dye in either sludge or silt, which still needs further treatment to remove the hazard^4–6^. In comparison, destructive methods, can directly decompose large dyes into non-toxic smaller molecules, which can be directly discharged into the environment. Of these destructive methods, photocatalytic reduction has emerged as the most popular. For example, bismuth oxychloride nanomaterials were synthesised via a hydrothermal method, and used for the degradation of Congo red with a 72% photocatalytic removal efficiency^7^. A Fe_2_O_3_–CeO_2_ nanocomposite prepared by a precipitation and improved sol gel self-ignition method, exhibited a 96% photodegradation removal efficiency for CR. A MgZnCr–TiO_2_ nanocomposite photocatalyst was also successfully synthesized by co-precipitation and removed 99.8% of CR after 180 min^8,9^. However, the disadvantage of these artificially synthesized catalysts was that the preparation process was complex and, also required expensive and toxic chemical reagents. The application of nanomaterials for photocatalysis is highly desirable due to the nanomaterial’s high surface areas and semiconductor heterogeneous structures, which significantly improves dye degradation efficiency. Although many nanoparticles have been reported for effective degradation, there are only a few studies which have considered the preparation of photocatalysts from waste materials. Shariffuddin et al.^10^ investigated the feasibility of using CaO derived from cockle shells as catalyst for MB removal and demonstrated good degradation. Likewise, E-CaO nanoparticles derived from waste eggshell were successfully prepared by a calcination method for efficient photodegradation of methylene blue (MB) and toluidine blue (TB)^11^, where the preparation of E-CaO was both eco-friendly and sustainable. These two studies clearly indicate that recycling wastes as nanomaterial remediating agents is certainly feasible. Hence, it is expected that in the ongoing search for more efficient and sustainable degradation methods for dyes, natural photocatalysts derived from waste biomass will provide an economical, sustainable and environmentally friendly method.

Clams are widely distributed in both the North and South Seas of China, where they grow rapidly, with a short breeding cycle and exhibit strong adaptability, thus making them an excellent shellfish suitable for artificial high-density cultivation. Clam shell is a significant by-product of this aquaculture, where not being fully utilized beneficially, results in large numbers of discarded clam shells being accumulated as biowaste. These discarded clam shells contain remnants of meat and other organic residuals, which will rot potentially producing toxins after long-term exposure to the environment, which has a negative impact on aquaculture, and also causes serious environmental pollution. Therefore, mishandling of by-products from aquaculture has previously led to an environmental crisis due to the unwanted release of hazardous substances^12^. Thus, an urgent waste issue to be resolved is how to effectively and beneficially utilize waste clam shells. Previously oyster shells have been used to effectively adsorb H_2_S from wastewater, with a maximum adsorption capacity of 12 mg/g^13^. Through calcination and hydration, waste oyster shell has also been pretreated to increase its specific surface area and pore volume, for the removal of SO_2_/NO_x_^14^. Oyster shell has also been used as an adsorption and filtration medium for phosphorus removal, where its use decreased eutrophication in wastewater^15^. Calcined mussel shells have also been used to remove mercury (Hg) from water with good efficiency, reaching 90% in 55 min^12^. A mixture of mussel shell calcined ash, sewage sludge and wood ash were used successfully as an adsorbent for several metal ions, with removal efficiencies for mercury (Hg), arsenic (As) and chromium (Cr) of is 98–99, 90–96, and 32%, respectively^16^. Many different natural materials have also been used to specifically remove anthropogenic dyes. The residual biomass of *Spirulina platensis* removed up to 82.6% of CR^17^, while carbonized kelp biochar performed even better, removing up to 94% of MB^18^. These reports indicate that waste utilization of marine aquaculture by-products has been an important issue. Even today, increasing attention has been directed towards better methods for the utilization of marine aquaculture by-products, as many studies have stressed the importance and necessity of global waste utilization. Thus, there seems to be great potential for the utilization of clam shell for cost-effective environmental pollution control. However, to the best of our knowledge, the potential for clam shell to act as a natural photocatalyst for dye degradation has not been previously studied, but this seems likely given the previous successes outlined above.

Thus, in this work, a natural photocatalyst based on calcium oxide derived from clam shell was synthesised via a high temperature calcination method and its physiochemical properties fully characterized by SEM, TEM, BET, XRD, XPS, UV–vis and TGA. Thereafter, the as-prepared photocatalysts were evaluated for their ability to adsorb and photocatalytically degrade double mixed cationic (MB) and anionic (CR) dyes, where the mechanism of adsorption and photocatalytic degradation was further explored.

## Materials and methods

### Materials

Clam shells were collected from a local market in Zhoushan, Zhejiang Province. Methylene blue (MB), Congo red (CR), sodium hydroxide, hydrochloric acid, p-benzoquinone, isopropanol, hydrogen peroxide and ammonium oxalate were all purchased from Shanghai Guoyao Chemical Reagent Co., Ltd. All chemicals used in this work were analytical grade and were used directly as received without further purification.

### Preparation of clam shell catalyst

Clam shells were initially washed with tap water to remove any obvious contamination from the surface of the clam shell. Thereafter, clam shells were immersed in a solution of 1 M sodium hydroxide and heated to 85 °C in a water bath remove any residual meat from the surface of the clam shells, then, the samples were rinsed with fresh water (pH = 7), and evaporated until dryness at 80 °C on a stove. The clam shells were soaked in 1 M hydrochloric acid solution in a clam pot overnight, washed with deionized water several times and dried. Finally, the clam shell was calcined in a muffle furnace for 2 h at 800, 900, and 1000 °C respectively. After calcination, the white powder obtained was ground, sieved (100 mesh) and stored in a desiccator for later use.

### Characterization

Specific surface area and pore size distribution of samples, was calculated using the BET method from N_2_ adsorption/desorption isotherms obtained using a static volumetric adsorption analyzer (Micromeritics ASAP 2010, Shanghai, China). Morphology and microstructure of the as-prepared samples was determined via a scanning electron microscope (SEM; S4800, Hitachi, Japan) and high-resolution transmission electron microscope (HR-TEM; Joel-2100, JEOL, Japan). Crystal structures were analyzed by X-ray diffraction (XRD, RIGAKU miniflex/600, Japan) in the range between 20° and 80°. Thermogravimetric analysis was conducted on a TGA Q5000 (American TA instrument Q Series) to better understand phase transformations during calcination. Optical properties of samples, were examined using a Cary 500 UV–vis NIR spectrophotometer in the range 200–800 nm. The chemical states of elements at the near surface of samples were also studied using X-ray photoelectron spectroscopy (XPS, Phi 5000C ESCA system, USA).

### Photocatalytic experiment

As-prepared photocatalyst (20 mg) was added into an aqueous solution of either MB or CR (40 mL) and the solution was magnetically stirred in the dark. for 2 h for the reaction to achieve adsorption equilibrium. During this time aliquots of the supernatant solution were removed at regular intervals (i.e., 0, 5, 10, 20, 30, 60, 90 and 120 min) an analyzed for the residual dye concentration. To assess adsorption performance, residual dye concentrations were determined photometrically.

After 2 h dark reaction, a light source (xenon lamp, HSX-F300) was used to initiate photocatalytic experiments. The vertical distance between the light source and the reactor was 10 cm, and supernatant samples were again taken at regularly intervals to assess photocatalytic performance. In the photocatalytic system, a cooling reflux device was also connected to the reaction vessel to eliminate the influence of heat generated by light on the experiment.

The effects of different dye concentrations (MB: 50, 75, and 100 ppm; CR: 400, 450, and 500 ppm), and different catalyst doses (20, 40, 60, and 80 mg) were also explored.

### Determination of dye concentration

After the reaction single dye solution samples were initially centrifuged at 4000 r min^-1^ for 10 min, and the absorbance of the supernatant subsequently measured using an UV–vis spectrophotometer. The wavelengths of absorbance maxima for the two dyes were λ_MB_: 664, and λ_CR_: 497 nm. The removal efficiency (R%) of adsorbed dye was calculated by Eq. (1), where the amount of dye adsorbed on the catalyst q_e_ (mg g^-1^) was calculated by Eq. (2).

1 $$ R({\text{\%) = }}\frac{{c_{0} - c_{e} }}{{c{}_{0}}} \times 100\% $$

2 $$q_{e} = \frac{{(C_{0} - C_{e} )V}}{m}$$

3 $$-\mathrm{ln}(\frac{{C}_{t}}{{C}_{0}^{^{\prime}}})=kt$$

where C_0_ and C_e_ (mg L^-1^) were respectively, the initial and equilibrium dye concentrations in solution, m (g) was the mass of photocataclyst and V (L) was the volume of dye solution. In order to study reaction kinetics, the rate constant k was obtained by curve fitting data to Eq. (3) where C’_0_ is the initial dye concentration after dark reaction and C_t_ is the dye concentration at light irradiation time t.

### Adsorption kinetics under dark reaction

Adsorption kinetics of the single dye system was further studied by adding catalyst to the dye solution under dark conditions with constant magnetic stirring and measuring the residual dye concentration in the solution at fixed time periods. The kinetic data so obtained was fit to both the pseudo first order (PFO Eq. 4) and second order (PSO Eq. 5) kinetics to evaluate the kinetics of adsorption.

4 $$\ln (q_{e} - q_{t} ) = \ln q_{e} - k_{1} t$$

5 $$\frac{t}{{q_{t} }} = \frac{1}{{k_{2} q_{e}^{2} }} + \frac{t}{{q_{e} }}$$

where q_t_ (mg g^-1^) is the dye adsorption capacity at time (t); and k_1_ (min^-1^) and k_2_ (g mg^-1^min^-1^) are the rate constants for PFO and PSO models, respectively.

## Results and discussion

### Characterization of catalyst

#### Morphology

**Figure 1:**
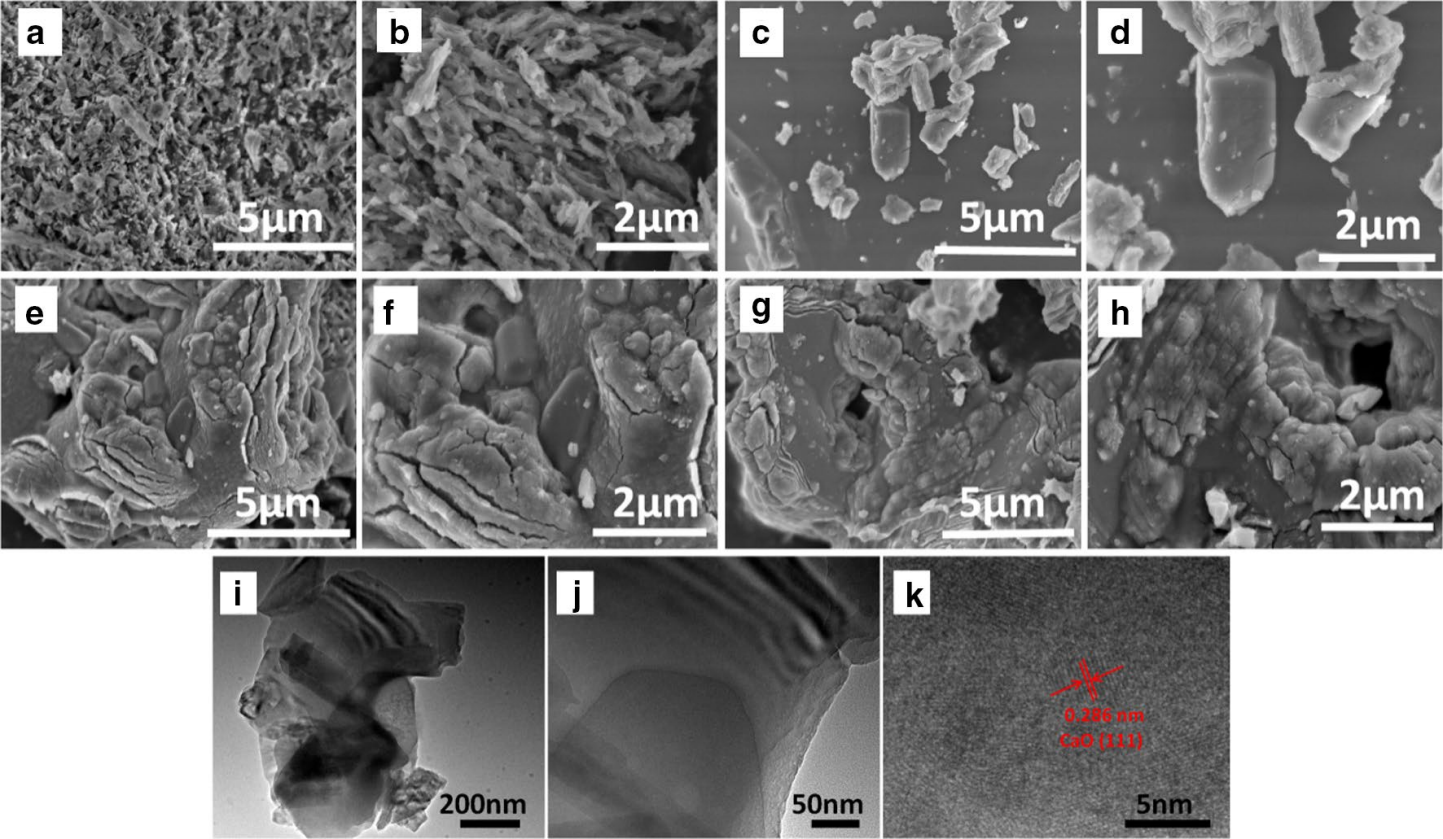
SEM images of clam shell samples: (**a**,**b**) uncalcined; (**c**,**d**) treated at 800 °C; (**e**,**f**) treated at 900 °C; (**g**,**h**) treated at 1000 °C; and TEM image (**i**–**k**) treated at 1000 °C.

The changes in clam shell powder morphology following calcination at three different temperatures were monitored using SEM and TEM (Fig. 1). Before calcination, clam shell powder had a spongy porous structure (Fig. a,b), due to presence of organic matter and a calcium carbonate skeleton after soaking in acid. However, after calcination at 800 °C, the clam shell surface became fragmented and bulky, due to the consumption of organic matter and growth of crystal particles (Fig. 1c,d). As calcination temperature further increased (900 and 1000 °C), clam shell powder morphology and surface structure became more compact. The increase in particle size was attributed to particle sintering, resulting in Ostwald ripening, which causes growth of larger particles at the expense of smaller ones. This was consistent with previous results which had shown an increase in average surface grain size with calcination temperature^19^.

More detailed morphological characteristics of clam shell calcined at 1000 °C were obtained by HR-TEM (Fig. 1i–k), and showed that after high temperature calcination the clam shell catalyst (CSC) had a layered structure where the distance between two adjacent lattices at the interface was 0.286 nm, which was consistent with XRD results (X-ray diffraction analysis) corresponding to the CaO (111) crystal face. In addition, the observed heterojunctions between dislocations in the crystal structure may be due to the rearrangement of additional atomic layers produced by the presence of trace metals in the CSC during calcination^20^. This may enable greater electron migration to the conductive band of CaO, since the energy band gap is reduced and charge carrier separation is increased, therefore improving photocatalytic performance.

#### BET analysis

All samples exhibited Type IV isotherms (Fig. S1 a and b) with hysteresis loops. The presence of small hysteresis loops at P/P_0_ > 0.4 indicated the existence of both microporous and mesoporous structures^21^. After calcination, specific surface area, pore volume and average pore diameter of the catalyst all decreased (Table S1). This was because as calcination temperature increased, the inner part of the particles shrunk more, leading to an overall decrease in the pore size of the material. However, specific surface area of the catalyst did increase slightly between 800 and 1000 °C, which was attributed to the formation of micro pores following decomposition of calcium carbonate. Adherence to the Type IV isotherm model clearly indicated the formation of multilayers on the catalyst surface during high temperature reactions, which was also clearly observed in TEM imagery (Fig. 1 i).

#### X-ray diffraction analysis

**Figure 2:**
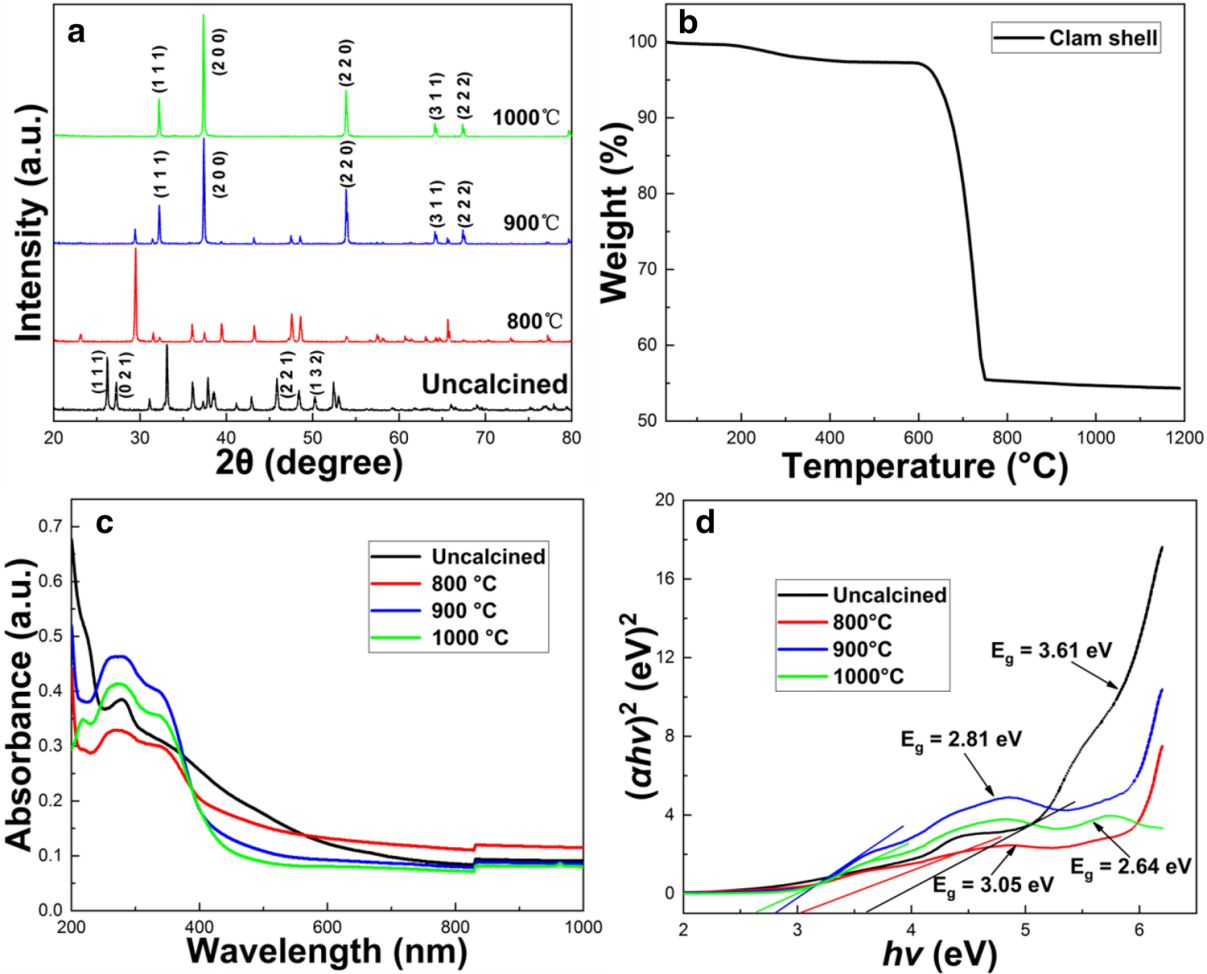
(**a**) XRD spectra of uncalcined, treated at 800, 900, and 1000 °C clam shell samples. (**b**) Thermogravimetric analysis of clam shell during calcination process. (**c**) Absorption spectrum and (**d**) optical band gap of different clam shell samples.

The XRD patterns of samples calcined at different temperatures (Fig. 2a), showed that the main component of untreated clam shell was CaCO_3_, where diffraction peaks at 2θ = 26.21°, 27.21°, 45.85° and 50.22° correspond to calcium carbonate on (111), (021), (221) and (132) crystal faces, respectively (PDF 70-1849). However, the XRD pattern of samples calcined at 800 °C for 2 h did not match well with either CaCO_3_ or CaO. This was because during heat treatment, calcium carbonate, the main component of the clam shell, would have experienced major structural changes from orthorhombic to trigonal-rhombohedral^11^. However, 800 oC is not enough for complete conversion, so the calcined material contains a large portion of intermediate phase/material between calcium carbonate and calcium oxide. For samples calcined for 2 h at either 900 or 1000 °C the diffraction peaks at 32.20°, 37.35°, 53.85°, 64.15° and 67.37° corresponded to calcium oxide on (111), (200), (220), (311) and (222) crystallographic planes, respectively^22^. In addition, closer comparison of the XRD patterns of the samples calcined at 900 °C and 1000 °C, showed that several small peaks present at 900 °C disappeared at 1000 °C. This was attributed to either further mass loss of trace metals or the complete conversion of intermediate substance to CaO. During calcination, calcium carbonate, the main component of clam shell, undergoes an initial phase change from aragonite to calcite, and then subsequently from calcite to CaO.

#### Thermogravimetric analysis

Thermogravimetric analysis provides further information on the transformation of clam shell powder during calcination. The initial mass lost from clam shell was only 0.47% due to the loss of water between 30–200 °C (Fig. 2b). Thereafter, the curve decreased slightly between 200–500 °C, due to the decomposition and removal of organic components of the clam shell, whereas weight loss between 500–610 °C was attributed to a phase transformation from aragonite to calcite. Thereafter, the mass of clam shell decreased significantly between 610 and 800 °C, due to further conversion of calcite to CaO and CO_2_, during calcination^10^. At 800–900 °C, further mass loss was caused by removal of any residual intermediate products from incomplete conversion of CaCO_3_ to CaO, and thereafter the slight decrease observed at 900–1000 °C, may be due to the presence of a small amount of unconverted CaCO_3_ and further mass loss of trace metals. All these results were in good agreement with both XRD (X-ray diffraction analysis) and XPS (XPS analysis) analysis.

#### Ultraviolet visible spectrum analysis

Diffuse reflectance spectroscopy in the UV–vis range was employed to investigate the optical properties of uncalcined clam shell powder and clam shell calcined at three different temperatures (Fig. 2c), as well as the light absorption characteristics and band gap energy of the CSCs (Fig. 2d). The wavelength absorption maxima of uncalcinated, 800, 900 and 1000 °C samples were 277, 271, 276 and 273 nm, respectively (Fig. 2c), corresponding to the optical band gap obtained from Tauc diagram (Fig. 2d). The optical band gap of the catalysts decreased with an increase in calcination temperature, and thus the lowest band gap (2.64 eV) was obtained at 1000 °C. As the calcination temperature increased, the weight of clam shell decreased, and the specific gravity of trace metal elements increased. Therefore, the decrease in optical band gap with increased calcination temperature was attributed to the appearance of localised energy states in the CaO band gap and the existence of a higher content of lattice defects, i.e., increases in trace metals and oxygen vacancies^23^. High resolution TEM (Morphology) had also confirmed the existence of a large number of lattice defects.

#### XPS analysis

**Figure 3:**
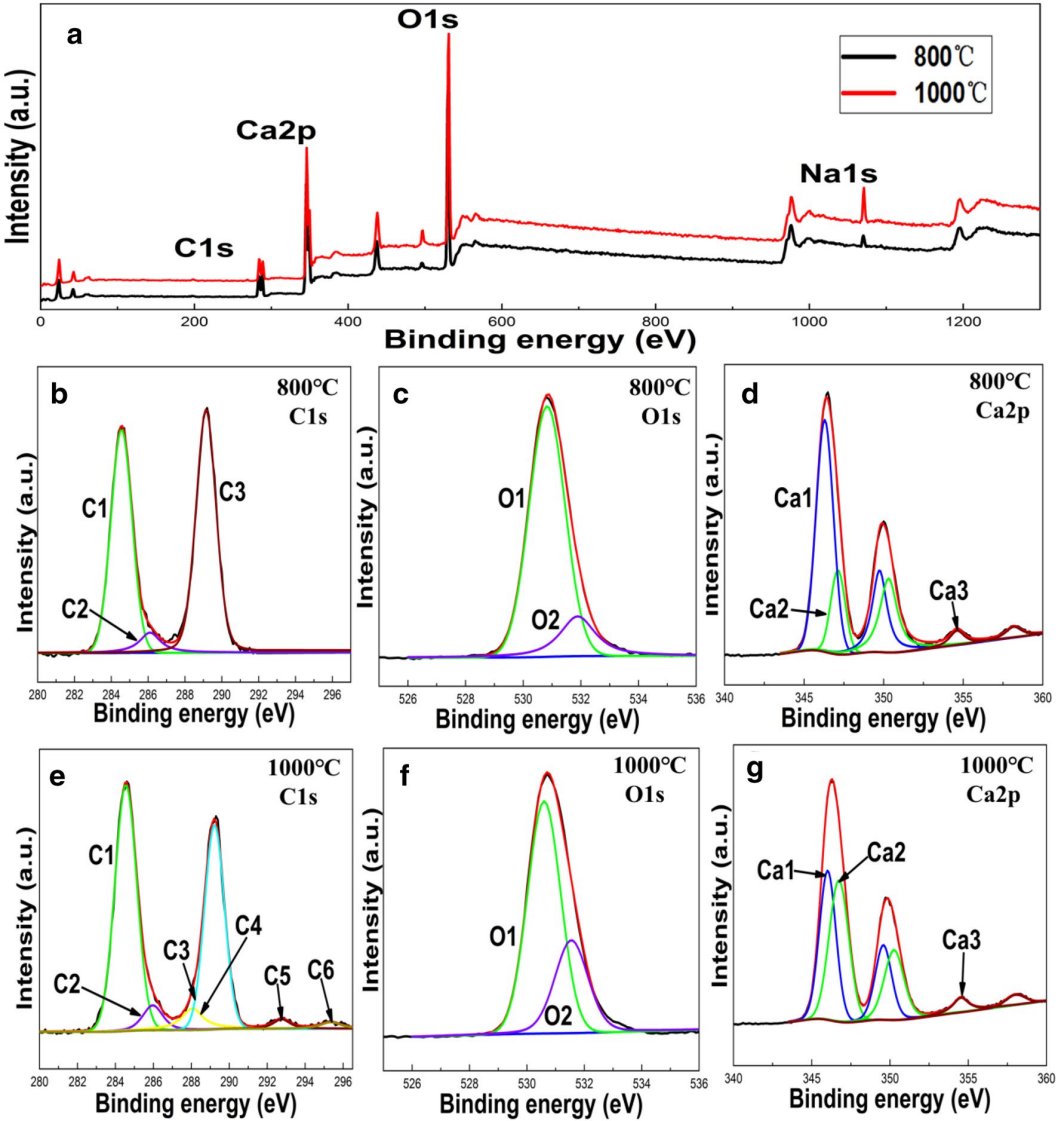
(**a**) XPS survey scan of CSCs at 800 °C and 1000 °C; XPS spectra of clam shell samples at: 800 °C (**b**) Ca2p, (**c**) C1s, (**d**) O1s, and 1000 °C (**e**) Ca2p, (**f**) C1s, (**g**) O1s of CSCs.

X-ray photoelectric spectroscopy (XPS) was used to analyze the valence state and content of surface elements in clam shell powder before and after high temperature (1000 °C) calcination, by comparing XPS spectra of clam shell treated at 800 and 1000 °C (Fig. 3). Survey scans (Fig. 3a), indicated that the main components of clam shells at 800 and 1000 °C were C, O, Ca and a small amount of Na. It is likely that traces of other metals such as magnesium (Mg) are also present but these would be at levels well below the detection limit of XPS (i.e., < 0.01%).

For clam shell powder calcined at 800 °C, the XPS spectra (Fig. 3b) exhibited many superimposed C1s peaks (C1–C3), where C1 with a binding energy of 285 eV, was attributed to amorphous carbon/adventitious carbon, a small standard C2 peak at 286.1 eV, corresponded to the C–O, and the C3 peak at 289.4 eV corresponded to a binding energy commonly associated with metal carbonate MCO_3_, and was thus attributed to CaCO_3_. In comparison, for clam shell calcined at 1000 °C (Fig. 3e), the binding energy of the adventitious carbon peak C1 occurred at 285 eV, the C2 peak for organic carbon (C–O bond) occurred at 286 eV, the C3 peak attributable to COOR at 288 eV, and the C4 peak corresponding to MCO_3_ at 289.4 eV. However, compared with the sample calcined at 800 °C, the sample calcined at 1000 °C, exhibited two extra weak peaks (C5 and C6) at 292 and 295 eV, respectively, which were attributed to compounds formed by carbon and/or halogen. The observed peak (O1) at 530.8 eV for the 800 °C calcined sample in the O1s XPS spectra was attributed to CO_3_^2–^ of CaCO_3_ (Fig. 3c); whereas the binding energy of the peak at 531.6 eV (peak O2) for the clam shell calcined at 1000 °C (Fig. 3f). corresponded to CaO.

Calcium typically exhibits two characteristic binding energies, i.e., Ca2p_3/2_ (346 eV) and Ca2p_1/2_ (349.6 eV)^24^. Here high-energy resolution spectra of Ca2p peaks showed a complex pattern that could be deconvoluted into three (Ca1, Ca2 and Ca3) constituent peaks (Fig. 3d,g). For sample calcined at 800 °C, peak Ca1 (346 eV), corresponded to CaCO_3_ and peak Ca2 (347.1 eV) was associated with CaO. In comparison, for the 1000 °C calcined sample the area of the CaO peak (Ca2) increased, indicating that the content of CaO was relatively larger when the sample was pyrolyzed at the higher temperature.

### Adsorption-photodegradation performance

#### Effect of different catalysts

**Figure 4:**
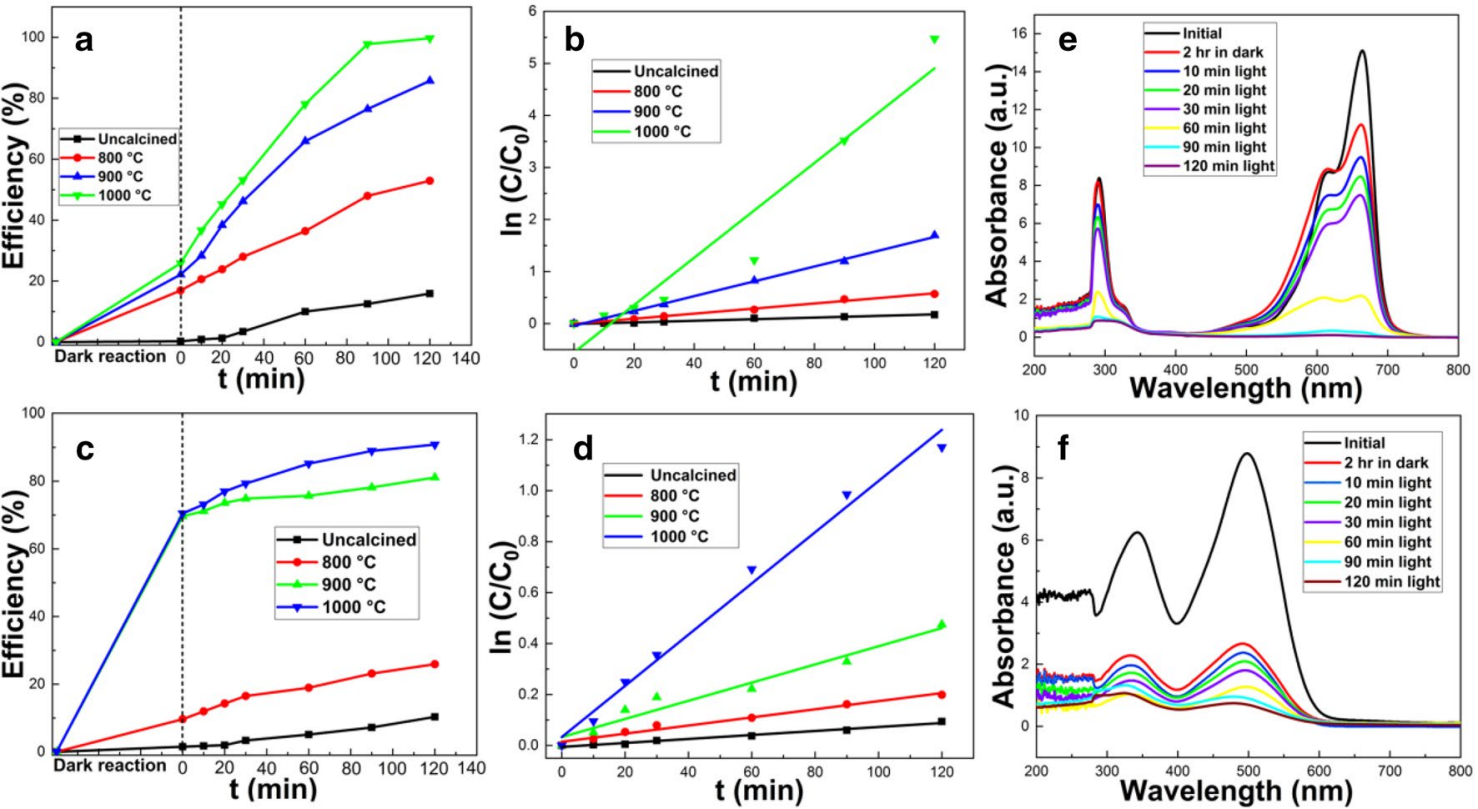
Photocatalytic experiments of MB (**a**,**b**,) and CR (**c,d**,) with calcined catalysts at different temperatures: (**a**,**c**) curve of removal rate with time, (**b**,**d**) plot of log of concentration with time. Photodegradation absorption spectra of (**e**) MB and (**f**) CR solutions in the presence of clam shell powder calcined at 1000 °C.

While the total adsorption-photodegradation removal efficiency of MB and CR for calcined clam shells at 1000 °C reached 99.7 and 90.8%, respectively, under dark conditions, the removal efficiencies of MB and CR reached only 25.9 and 70.5%, respectively (Fig. 4). In comparison the total absorption-photodegradation removal efficiency of untreated clam shells (MB: 15.9%, CR: 10.4%), and those calcined at 800 °C (MB: 53.9%, CR: 25.9%), or 900 °C, (MB: 85.7%, CR: 81.1%) were lower. This indicated that the adsorption and/or photocatalytic properties of uncalcined clam shell or clam shell calcined at temperatures < 1000 °C were poorer than clam shell calcined at 1000 °C. It can also be clearly seen (Fig. 4) that the adsorption performance (dark environment) of clam shell calcined at different temperatures was consistently better than that of non-calcined clam shell. This suggested that decomposition of residual organic matter in the original clam shell via heat treatment during calcination, results in significantly more pore structure. Where the increase in adsorption performance with calcination temperature, could be attributed to increased pore forming effects from CO_2_ generated by thermal decomposition of CaCO_3_, the main component of clam shell. The photocatalytic activity (under light) of the clam shell powder was also enhanced by an increase in calcination temperature. This may be due to an increase in the relative content of calcium oxide due to heating and the interaction of trace transition metals in the clam shell. HR-TEM images confirmed the presences of a significant number of dislocations and defects in the CaO crystal structure. Overall, results from the adsorption-photodegradation study indicated that the best photocatalytic performance was obtained when clam shell powder was calcined at 1000 °C. This was attributed to synergetic effects of enhanced absorption and increased heterojunctions among some dislocations in the crystal structures, which also improved photodegradation efficiency.

Photodegradation kinetics of both MB and CR by CSCs varied with the calcination temperature of the catalyst (Fig. 4b,d). The kinetic data for the degradation of both MB and CR best fit the pseudo first order kinetic model (R^2^ approaching 1), where the rate constant (k) increased with calcination temperature (Table 1), and the higher the k value, the better the photocatalytic performance and the greater the photodegradation amount. Thus, in all subsequent experiments, only clam shell calcined at 1000 °C was investigated.

**Table 1:** Best fit photodegradation kinetic parameters for MB and CR with the four clam shells studied.

Dye	Sample	Uncalcined	800 °C	900 °C	1000 °C
MB	R^2^	0.9733	0.9926	0.9973	0.9211
	k	0.0015	0.0049	0.0142	0.0455
CR	R^2^	0.9779	0.9762	0.9509	0.9869
	k	0.0008	0.0016	0.0036	0.0101

The photodegradation of both MB and CR by 1000 °C calcined CSC occurs in the visible light region of the absorption spectrum (Fig. 4e,f). As reaction time increased, peaks of MB and CR both decreased sharply, and some peaks disappeared or were slightly shifted in wavelength, which indicated that both MB and CR molecules were being destroyed, and that new substances (degradation products) were also formed^10^.

#### Adsorption performances

The adsorption performance of 1000 °C calcined clam shell powder varied with the initial concentrations of MB and CR within 2 h. (Fig. S2) Over 2 h the concentration of both MB or CR decreased gradually after exposure to the CSC until the adsorption equilibrium was reached. When the initial dye concentration was low, equilibrium was reached relatively quickly, within 30 min. For example, for MB a sharp decrease in MB concentration was observed in the first 10 min followed by a slower adsorption until the MB concentration stabilized. This is because when the initial dye concentration is low, the adsorption reaction can quickly reach equilibrium because there are a large number of sites available on the surface of CSC for adsorption relative to the number of MB molecules. As the initial concentration of dye increases, a more competitive adsorption for MB molecules for surface sites is induced. Thus, as more active sites become occupied, it becomes increasingly more difficult to adsorb dye due to a weaker attraction between the surface of CSC and the MB molecules, which leads to slowing of dye adsorption rate in the latter stages of the reaction.

In order to further investigate the adsorption mechanism of the MB dye on to the CSC, both pseudo first order (PFO) (Eq. 4) and pseudo second order (PSO) (Eq. 5) kinetic models were used for data analysis. The kinetic data for MB adsorption on to CSC fit both the PFO (Fig S2(c)) and PSO (Fig. S2(d)) models well with good linear behavior. However, the goodness of fit (R^2^) for the PSO model was slightly higher than that for the PFO model, and the adsorption capacity at equilibrium obtained from PSO fitting was also closer to the experimental value (Table S2). The corresponding experimental adsorption capacities for initial MB concentrations of 50, 75 and 100 ppm were 60.52, 96.72 and 123.45 mg g^-1^, respectively. Therefore, the PSO model was more suitable for describing dye adsorption to CSC. Based on the underlying assumptions of the PSO equation model this indicates that MB participated in chemical adsorption, involving the sharing or exchange of electrons between the hydrophilic edge sites of the CSC and the dye cations^25^. As the initial MB concentration increased, the goodness of fit of adsorption capacity (q_e_) also increased, and became very close to the experimentally derived q_e_ value, but the adsorption rate (k_2_ value) decreased. This decrease in rate constant is due to the intense adsorption competition between relatively limited surface-active sites and a large number of MB molecules in a higher concentration dye solution, resulting in a relatively low-rate constant^26^.

Similarly, the initial adsorption capacities for CR at 400, 455 and 500 ppm were 592.76, 653.26 and 679.91 mg g^-1^, respectively where fitting of the kinetic data to the same two models (Fig. S2 (g) and (h)) showed that the PSO model better described the adsorption of CR on the CSC, with a calculated adsorption amount at equilibrium closer to the experimental value (Table S2). Like MB, since the PSO model assumes chemical adsorption as the dominant adsorption process^27,28^, which involves the sharing or exchange of electrons between the hydrophilic edge sites of the CSC and the dye ions, this result also suggests that negatively charged CR ions were removed from solution via a chemical adsorption process. This adsorption may be caused by a combination of π–π stacking, hydrogen bonding, and van der Waals forces. In addition, the k_1_ values for CR were much lower than those calculated for MB, which indicated that the CSC had a higher affinity for the negatively charged dye^29^.

#### Effect of catalyst dose

The catalyst dose is an important practical consideration and also plays an important role in determining the efficiency of the photocatalytic reaction. An increase in catalyst dose will increase the overall concentration of active substance and hence increase the number of active sites in the reaction system. Thus, the probability of contact of dye molecules with the active sites increases, as does the reaction probability, and rate of reaction, leading to improved dye removal efficiency. For photodegradation removal processes there will generally be an increase in removal efficiency with an increase in dose until, at a certain concentration, the increase in catalyst amount reduces the light transmittance of the suspension^30^ and hence the removal efficiency.

**Figure 5:**
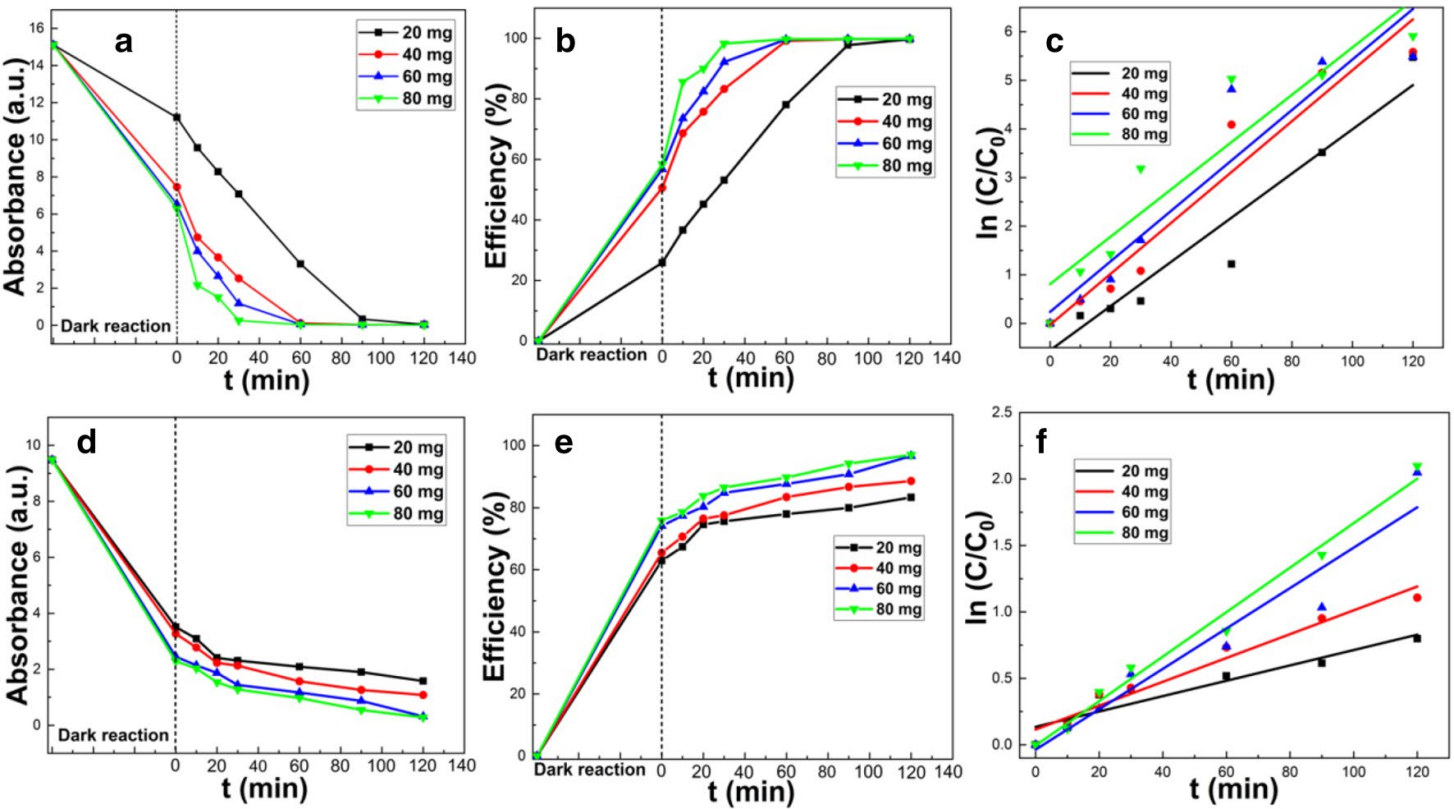
Photocatalytic experiment of MB (**a**–**c**) and CR (**d**–**f**) with different doses of clam shell at 1000 °C (**a,d**) curve of absorbance with time, (**b**,**e**) curve of removal rate with time, (**c**,**f**) plot of log of concentration with time.

For MB and CR (Fig. 5) as the amount of CSC increased from 20, 40, 60 to 80 mg, the removal efficiencies of MB were 99.7, 99.8, 99.9 and 99.9%, respectively, and 83.3, 88.6, 96.7% and 97.0% for CR, respectively. Thus, for both dyes’ removal efficiency increases with catalyst dose, being almost a negligible for MB and only modest for CR. This indicated that even the lowest dose used here had an adsorption capacity well above the amount of dye in solution. Even though the increase in MB removal efficiency with dose was not very significant, the time required to achieve this removal became less as dose increased (Fig. 5b). Since even a 20 mg catalyst dose could achieve a strong catalytic effect, in 40 mL dye solutions a catalyst dose of 0.5 g L^-1^ was selected for all subsequent experiments. According to the experimental data, enough binding sites can be reached at 20 mg. Although the removal efficiency further increases with an increase in the amount of catalyst, the higher concentration is not essential for performance and are thus a waste of catalyst, since nearly all of the additional binding sites are not used.

The best fit kinetic parameters (Table 2) for MB and CR degradation under different catalyst doses showed that the degradation of both MB and CR followed pseudo first order kinetics, with good correlation (R^2^ close to 1).

**Table 2:** Variation in best fit degradation kinetic parameters for MB and CR with the CSC dose.

Dye	Catalyst concentration	20 mg	40 mg	60 mg	80 mg
MB	R^2^	0.9211	0.9332	0.8797	0.8541
	k	0.0455	0.0524	0.0519	0.0487
CR	R^2^	0.9562	0.9531	0.9228	0.9839
	k	0.0058	0.0090	0.0152	0.0167

The k value for MB increased slightly in the range of 20–40 mg, and decreased between 40 and 80 mg. This was potentially because the increase of catalyst above 40 mg reduced the light transmittance of the suspension, making it more difficult for photons to reach the CSC surface, and the amount of •OH free radical formed between dye molecules and catalyst surface became relatively small. However, for CR, the k value increased significantly between 20 and 60 mg, and slightly increased from 60 to 80 mg.

#### Effect of initial dye concentration

**Figure 6:**
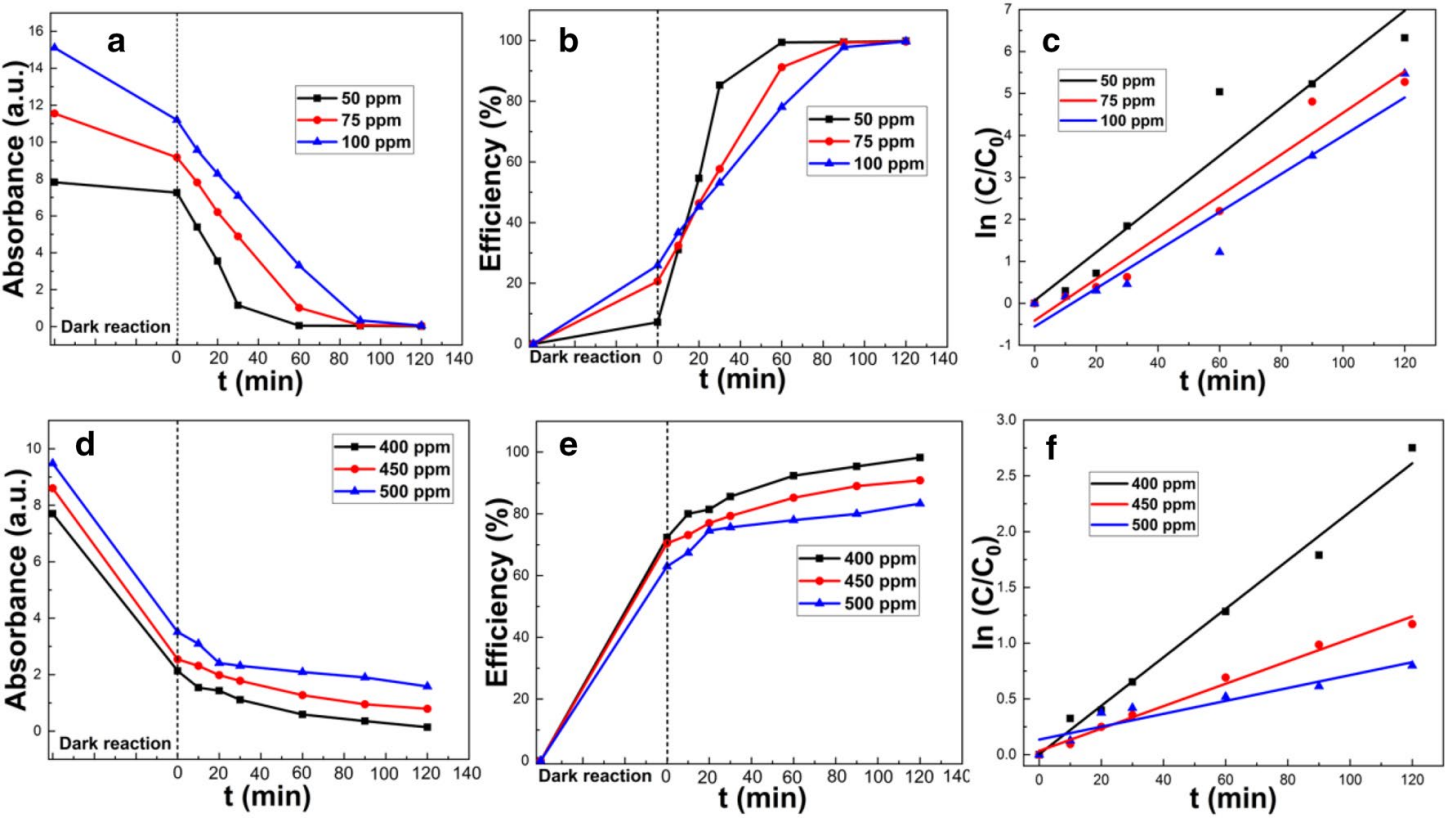
Photocatalytic experiment of clam shell at 1000 °C for MB (**a**–**c**) and CR (**d**–**f**); Curves of absorbance with time (**a**,**d**); Curves of removal rate with time (**b**), (**e**); Plots of log of concentration with time (**c**,**f**).

The removal efficiencies of MB dye at initial dye concentrations of 50, 75 and 100 ppm were 99.8, 99.7 and 99.6%, respectively (Fig. 6). Although the removal efficiency did not significantly change with initial dye concentration, the time required for the photodegradation become longer (Fig. 6). Likewise, after 120 min of illumination, the overall removal efficiency of CR dye decreased with an increase in the initial dye concentration, being 98.2, 90.8 and 83.3%, for CR concentration of 400, 450 and 500 ppm respectively. This is because the number of •OH radicals formed on the catalyst surface and the number of interactions with dye molecules determine the overall efficiency of photocatalytic degradation. With an increase in the number of dye molecules in solution, the number of dye molecules contacting the catalyst surface also increases, causing active sites on the catalyst surface to be covered by them, which will affect the arrival of photons on the surface of the catalyst, and will reduce the catalytic efficiency, resulting in less •OH radical generation on the catalyst surface. Therefore, increased dye concentration can lead to a reduction in both degradation efficiency and reaction rate^31^.

The variation in best fit degradation kinetic parameters for both MB and CR with different concentrations are shown in Table 3. The k values of both MB and CR increased as the initial dye concentration decreased. The maximum k value of 0.058 min^−1^ was observed for MB, which demonstrated that this catalyst had the highest photocatalytic degradation rate for MB. This was also much higher than result obtained using other semiconductors such as TiO_2_/MoS_2_ with k = 0.040 min^−1^^32^, g-C_3_N_4_/MnV_2_O_6_ (1:1), k = 0.022 min^−1^^33^,CaTiO_3_:0.5%Eu^3+^, k = 0.005 min^−1^^34^, and Bi_2_CrO_6_, k = 0.006 min^−1^^35^.

**Table 3:** Variation in best fit degradation kinetics parameters for MB and CR with initial dye concentration.

Dye	Dye concentration	50 ppm	75 ppm	100 ppm
MB	R^2^	0.912	0.953	0.921
	k	0.058	0.049	0.045
CR	Dye concentration	400 ppm	450 ppm	500 ppm
	R^2^	0.987	0.986	0.856
	k	0.022	0.010	0.006

#### Influence of free radical scavengers on catalyst performance

**Figure 7:**
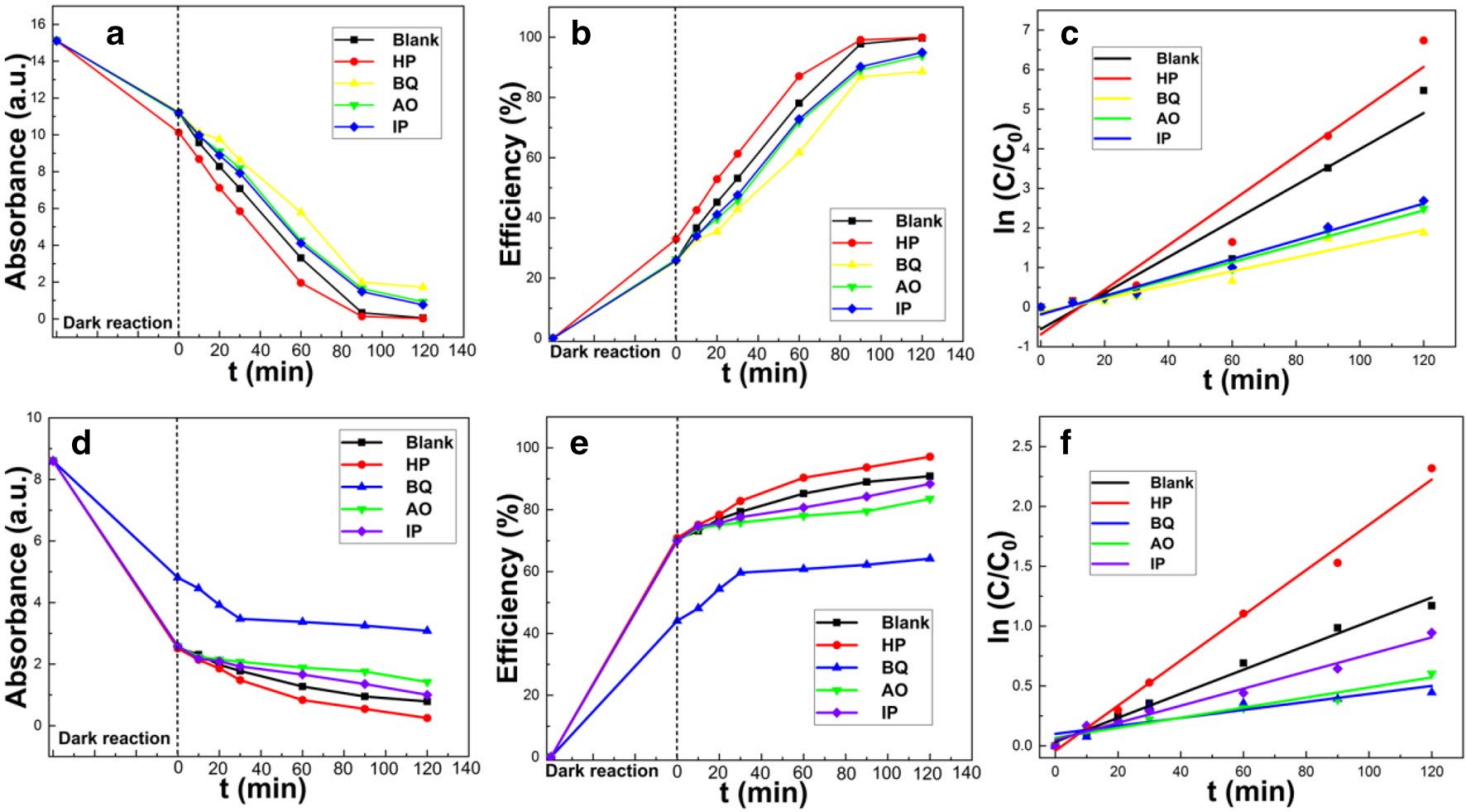
Effects of scavenger and superoxide on MB (**a**–**c**) and CR (**d**–**f**) degradation.

Since the photodegradation efficiency of the catalyst is likely to depend on the concentration of free radicals at the surface, free radical scavengers can affect photodegradation performance. Here, in order to examine the factors affecting the performance of the catalyst, isopropanol (IP) as a •OH radical scavenger, p-benzoquinone (BQ) as an •O_2_^-^ radical scavenger, and ammonium oxalate (AO) as a hole (h^+^) scavenger were added to the reaction system^36,37^. Under illumination from a xenon lamp, the removal efficiencies of MB with BQ, AO and IP were 88.6%, 93.9% and 95.0%, respectively (Fig. 7) compared to a MB removal efficiency of 99.9 with no added scavengers. Similarly, the removal efficiencies for CR after adding BQ, AO and IP were 64.9, 83.5 and 88.4% respectively, compared to 90.8% without any added scavenger.

The presence of BQ, significantly inhibited the removal efficiencies of both MB and CR This was because BQ leads to a decrease in the amount of superoxide radicals present, which leads to a significant decrease in the photodegradation rate (Table 4). In comparison, AO and IP induced only slight decreases in the removal efficiencies of MB and CR. These experimental results imply that while the formation of •O_2_^−^ and h^+^ in the photocatalytic reaction are both essential for a high removal efficiency, •O_2_^−^ is the dominant reactive species in this reaction.

**Table 4:** Variation in best fit degradation kinetic parameters of MB and CR in the presence different scavengers and superoxide.

Dye	Reagent	Blank	HP	BQ	AO	IP
MB	R^2^	0.921	0.931	0.937	0.976	0.976
	k	0.045	0.056	0.017	0.022	0.023
CR	R^2^	0.986	0.991	0.884	0.933	0.978
	k	0.010	0.019	0.003	0.004	0.007

These observations allow a removal mechanism to be proposed. When the CSC is irradiated by a xenon lamp, h^+^ electron holes are generated in the collision gap. Oxygen molecules in the mixed solution form •O_2_^−^ superoxide radicals by electron removal, and h^+^ react with water to form •OH radical^38^.

The addition of hydrogen peroxide (H_2_O_2_) is often used as an amendment to enhance photocatalytic degradation of organic materials by enhancing free radical generation. Here, under H_2_O_2_ assisted photocatalysis, the removal efficiencies of MB and CR were 99.9% and 97.1%, respectively, indicating that compared to the blank samples (99.6% and 90.8%), the addition of H_2_O_2_ significantly improved the photodegradation removal efficiency. The reaction rate constants of the two dyes were also close to each other, indicating that these two dyes were removed via similar mechanisms.

H_2_O_2_ assisted photocatalysis works through the formation of hydrogen peroxide ion (HO_2_^−^) via reaction with H_2_O, accelerating the hydrolysis of H_2_O_2_ forming OH radical, and is accompanied by the formation of hydrogen hydrate ion (H_3_O^+^) ^39^, and the further reaction of HO_2_^-^ and H_2_O_2_ forms OH radical, which is the decisive step of the degradation rate of organic pollutants in aqueous solution^40^. The reaction mechanism is summarized as follows:

6 $${\text{H}}_{{2}} {\text{O}}_{{2}} + {\text{H}}_{{2}} {\text{O}} \to {\text{ H}}_{{2}} {\text{O}}^{ - } + {\text{H}}_{{3}} {\text{O}}^{ + }$$

7 $${\text{H}}_{{2}} {\text{O}}_{{2}} + {\text{ HO}}_{{2}}^{ - } \to {\text{OH}}\cdot \, + {\text{ HO}}_{{2}} \cdot \, + {\text{ OH}}^{ - }$$

In the process of photocatalytic degradation of dyes, the electrons generated by the catalyst can react with O_2_ to form •O_2_^−^, and then the products can react with water to form hydrogenated oxygen radicals (HO_2_•) and hydroxyl radicals (OH^-^). The generated HO_2_• rearranges to form oxygen (O_2_) and peroxide hydrogen (H_2_O_2_)^41^, and H_2_O_2_ reacts with superoxide radical to form hydroxyl radical (OH^-^). The generated h^+^ interacts with water to form a highly reactive hydroxyl radical, the hole itself can also attack dye molecules to produce by-products as well. After the formation of free radicals, the reactive substance (•O_2_^−^) attacks dye molecules until the end of decolourisation and ring opening reaction, effectively degrading the dye into smaller intermediates and the final products (CO_2_ and H_2_O)^42–44^. The possible photocatalytic reactions are as follows:

8 $${\text{CaO }} + {\text{ hv}} \to {\text{ h}}^{ + } \, + {\text{ e}}^{ - }$$

9 $${\text{e}}^{ - } \, + {\text{ O}}_{{2}} \to \, \cdot{\text{O}}_{{2}}^{ - }$$

10 $$\cdot{\text{O}}_{{2}}^{ - } + {\text{ H}}_{{2}} {\text{O }} \to {\text{OH}}^{ - } + {\text{ HO}}_{{2}} \cdot$$

11 $${\text{2HO}}_{{2}} \cdot \, \to {\text{O}}_{{2}} + {\text{ H}}_{{2}} {\text{O}}_{{2}}$$

12 $${\text{H}}_{{2}} {\text{O}}_{{2}} + \, \cdot{\text{O}}_{{2}}^{ - } \to {\text{O}}_{{2}} + {\text{ OH}}^{ - } + \, \cdot{\text{OH}}$$

13 $${\text{h}}^{ + } + {\text{ H}}_{{2}} {\text{O }} \to \cdot{\text{OH }} + {\text{ H}}^{ + }$$

14 $${\text{Dye }} + {\text{ hv}} \to {\text{ Dye}}^{*}$$

15 $${\text{Dye}}^{*} \, + \left( {\cdot{\text{OH}}, \, \cdot{\text{O}}_{{2}}^{ - } ,{\text{ h}}^{ + } } \right) \to {\text{ Intermediates }} \to {\text{CO}}_{{2}} + {\text{ H}}_{{2}} {\text{O}}$$

### Proposed photocatalytic mechanism

Based on the above experimental studies, a possible mechanism for the photocatalytic degradation of dyes by the CSC produced here was proposed (Fig S3). Under light irradiation, the electrons gain sufficient energy to become excited and transition from the valence band to the conduction band, and form excitons (electron–hole pairs). These excitons are known to act in four potential ways: (1) intralattice recombination, (2) recombination at surface active sites, (3) hole (h^+^) oxidizes external dyes, and (4) electron (e^-^) reduces external dyes. High photocatalytic activity can be achieved through the minimum photocatalytic system of electron hole recombination, and the combination of effective absorption and enhanced electron transition from heterojunctions among some dislocations in the crystal structures^45,46^. It was previously reported that photocatalytic degradation efficiency can also be further improved via effective size reduction of CaO nanoparticles and the innate nano properties of the initial raw materials^47^. Hence, calcium oxide nanoparticles synthesized by calcination of marine biomass, such as shells or other biomass materials containing metal oxides and trace transition metals, which can act as excellent natural photocatalysts for the degradation of dyes from the textile industries. In contrast, while the photodegradation efficiency of 50 mg CaO derived from eggshell biomass catalyst can reach 96.2% at an initial MB dye concentration of 20 mg/L through parameter optimization^11^, here, the photodegradation efficiency using a 20 mg CSC derived from marine biomass reached 99.6% for an initial MB dye solution concentration of 100 mg/L.

### Feasibility of practical application

In the textile industry, dye wastewater typically contains many kinds of different dyes which need to be simultaneously removed. Hence here, the feasibility of applying a CSC for practical wastewater treatment was evaluated in a mixed dye solution. The results indicated that 20 mg of CSC could easily degrade both a mixed dark colored binary dye solution (100 ppm MB + 100 ppm CR) and a ternary dye system (75 ppm MB + 75 ppm CR + 75 ppm rhodamine B (Rh B)) into a transparent solution (Fig S4). Analysis of the absorption spectra of the mixed dye solution system under visible-light irradiation, showed that the initial peaks of the mixed dye solution decreased sharply as reaction time increases, with some peaks shifting slightly or completely disappearing, which strongly suggested that mixtures of dye molecules were destroyed or degraded, and new substances were formed.

## Conclusion

A novel photocatalyst nanomaterial derived from natural clam shell was successfully prepared via a facile calcination process. The as prepared material was then evaluated for its potential to treat dye contaminated wastewater. Under xenon lamp illumination, the overall absorbance- photocatalytic removal efficiencies for 100 mg L^-1^ MB and 500 mg L^-1^ CR reached 99.6 and 83.3%, respectively. Increasing the calcination temperature enhanced the catalytic performance of the catalyst. This was attributed to synergetic effects of increased absorption together with increased heterojunctions among some dislocations in the crystal structures. Increased numbers of heterojunction can greatly reduce the energy required for electron transition, making it much easier to form holes and excited electrons. Consequently, the inexpensive clam shell photocatalyst, synthesized in this work from a marine biowaste, may have great potential for future practical large-scale industrial wastewater treatment applications.

## Supplementary Information

Supplementary Information.
