# Supplementary material for: A sustainable natural clam shell derived photocatalyst for the effective adsorption and photodegradation of organic dyes
Source: Sci Rep. 2022 Feb 22;12:2988. doi: 10.1038/s41598-022-06981-3 (PMC8863817; doi:10.1038/s41598-022-06981-3)
Supplement: Supplementary file 1 — Supplementary Information. [file 41598_2022_6981_MOESM1_ESM.docx]

**Supplementary Materials**

**
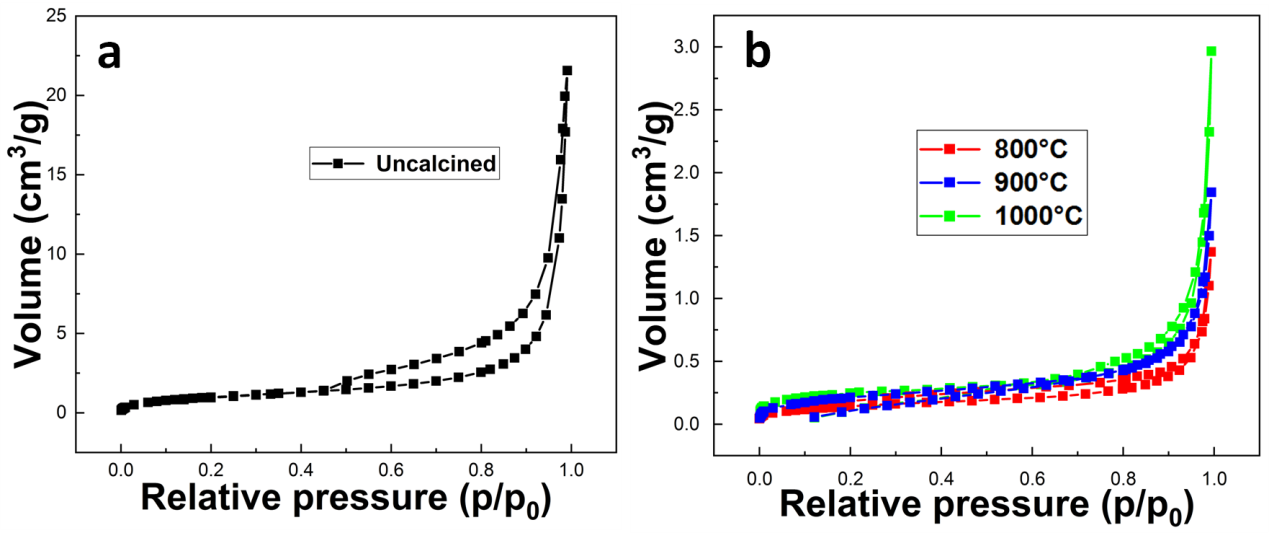
**

**Fig. S1.** Adsorption and desorption isotherms of (a) uncalcined; (b) calcined at 800, 900 and 1000 °C CSCs.


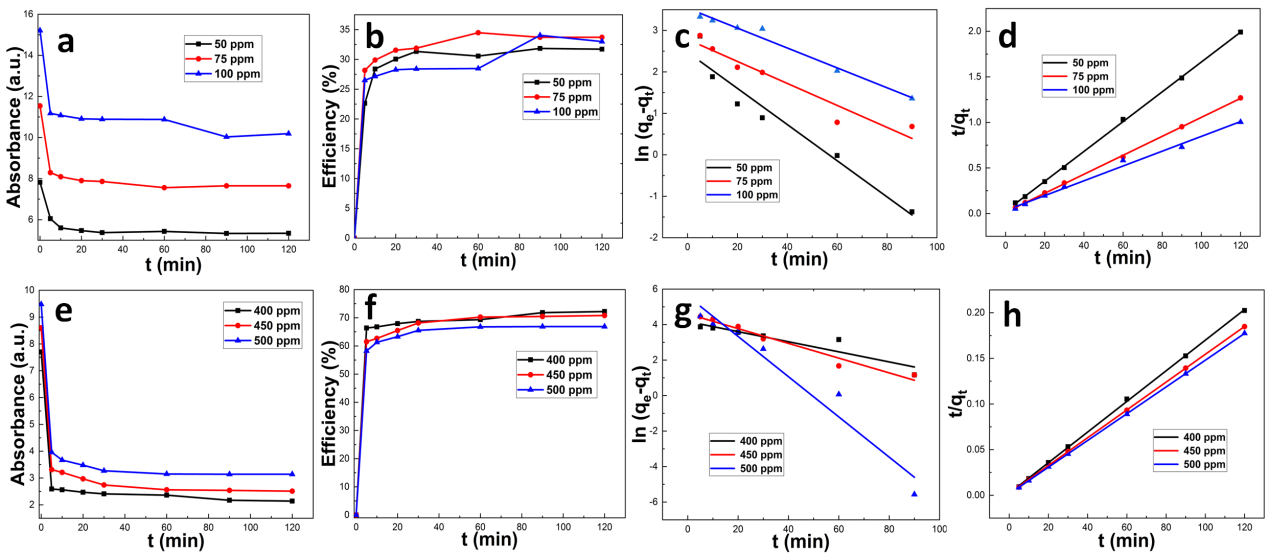


**Fig. S2.** Adsorption experiments of MB (a-d) and CR (e-h) on clam shell at 1000 °C: (a), (e) adsorption at different concentrations; (b), (f) adsorption rates at different concentrations; (c), (g) pseudo first order kinetics; (d), (h) pseudo second order kinetics.


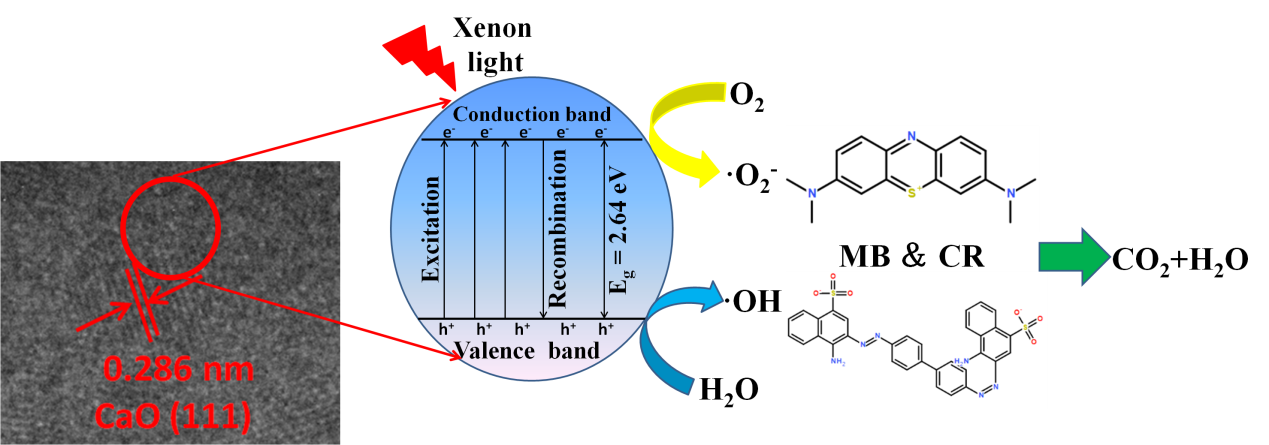


**Fig. S3.** Mechanism scheme of photocatalytic degradation of dyes by CSC.


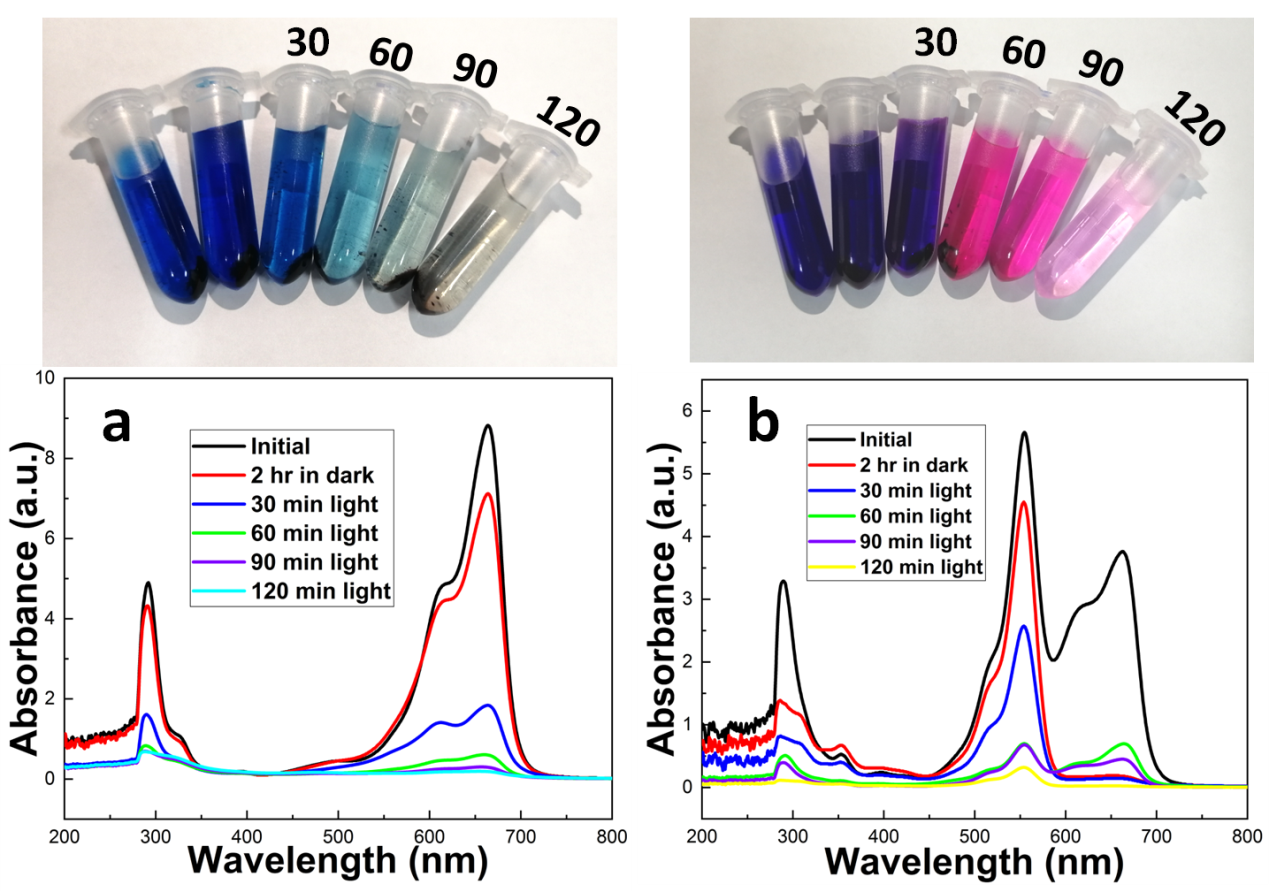


**Fig. S4.** Photodegradation of a mixed dye by clam shell powder at 1000 °C, (a) binary system (100ppm MB + 100ppm CR), (b) ternary system (75ppm MB + 75ppm CR + 75ppm Rh B).

**Table S1.** Texture characteristics of uncalcined and calcined CSCs.

| Sample | S_BET_ (m^2^ g^-1^) | V_tot_ (cm^3^ g^-1^) | Average pore diameter (nm) |
| --- | --- | --- | --- |
| Uncalcined | 3.76 | 0.010 | 10.98 |
| 800°C | 0.51 | 0.001 | 6.28 |
| 900°C | 0.76 | 0.001 | 6.33 |
| 1000°C | 0.97 | 0.002 | 6.77 |

**Table S2.** Best fit kinetic parameters for adsorption of MB and CR onto clam shell calcined at 1000 °C.

| Dye |  | Pseudo-first-order | | | Pseudo-second-order | | |
| --- | --- | --- | --- | --- | --- | --- | --- |
|  | Values | q_e_ (mg g^-1^) | k_1_ (min^-1^)  ×10^-3^ | R^2^ | q_e_ (mg g^-1^) | k_2_ (g mg^-1^)  (min^-1^) ×10^-3^ | R^2^ |
| MB | 50 ppm | 11.88 | 43.63 | 0.928 | 61.09 | 10.20 | 0.999 |
|  | 75 ppm | 16.17 | 26.53 | 0.902 | 95.97 | 7.88 | 0.999 |
|  | 100 ppm | 34.32 | 23.99 | 0.974 | 123.0 | 1.96 | 0.990 |
| CR | 400 ppm | 64.81 | 28.44 | 0.825 | 595.2 | 1.33 | 0.999 |
|  | 450 ppm | 99.89 | 21.55 | 0.956 | 657.8 | 1.12 | 0.999 |
|  | 500 ppm | 269.7 | 11.33 | 0.943 | 680.2 | 1.05 | 0.999 |
